# Neuropathic Pain in the Elderly

**DOI:** 10.3390/diagnostics11040613

**Published:** 2021-03-30

**Authors:** Silvia Giovannini, Daniele Coraci, Fabrizio Brau, Vincenzo Galluzzo, Claudia Loreti, Pietro Caliandro, Luca Padua, Giulio Maccauro, Lorenzo Biscotti, Roberto Bernabei

**Affiliations:** 1 Department of Aging, Neurological, Orthopaedic and Head-Neck Sciences, Fondazione Policlinico Universitario Agostino Gemelli IRCCS, 00168 Rome, Italy; danielecoraci@aol.com (D.C.); febrisb@gmail.com (F.B.); v91galluzzo@gmail.com (V.G.); claudia.loreti@policlinicogemelli.it (C.L.); pietro.caliandro@unicatt.it (P.C.); luca.padua@unicatt.it (L.P.); giulio.maccauro@unicatt.it (G.M.); lorenzo.biscotti@unicatt.it (L.B.); roberto.bernabei@unicatt.it (R.B.); 2 Department of Neurosciences, Università Cattolica del Sacro Cuore, 00168 Rome, Italy; 3 Department of Geriatrics and Orthopaedics, Università Cattolica del Sacro Cuore, 00168 Rome, Italy; 4 Presiding Officer of Geriatric Care Promotion and Development Centre (C.E.P.S.A.G), Università Cattolica del Sacro Cuore, 00168 Rome, Italy

**Keywords:** neuropathic pain, aging, personalized medicine

## Abstract

Neuropathic pain due to a lesion or a disease of the somatosensory system often affects older people presenting several comorbidities. Moreover, elderly patients are often poly-medicated, hospitalized and treated in a nursing home with a growing risk of drug interaction and recurrent hospitalization. Neuropathic pain in the elderly has to be managed by a multidimensional approach that involves several medical, social and psychological professionals in order to improve the quality of life of the patients and, where present, their relatives.

## 1. Introduction

Neuropathic pain in the elderly is a common but unrecognized clinical issue. In the general population, recent surveys reported prevalence rates of between 6.9% and 10% for neuropathic pain [1], while data on the prevalence among older people are scarce. Due to cognitive impairment and concurrent illnesses, older people often underreport pain, especially to primary care physicians [2]. Moreover, aging reveals anatomical and biological changes, such as loss of neurons in the central nervous system, increased number of abnormal or degenerating fibrers, slower conduction velocity, altered endogenous inhibition and decreased function of neurotransmitters [3,4,5]. These anatomical changes are involved in the altered perception of neuropathic pain among older people. Finally, difficulties in conducting questionnaires among patients with dementia or visual and hearing disorders could delay the diagnosis of neuropathic pain.

Despite the age-related organic changes, both younger and older people might be affected by the same chronic diseases which carry on the common manifestations of neuropathic pain. This explains why classification of different types of neuropathic pain and first clinical approach do not differ between all ages.

If reported, pain mostly results from the stimulation of pain receptors. This kind of pain is called nociceptive pain, and its treatment is based on common analgesic medications [3,6]. Neuropathic pain is often persistent and more difficult to treat than nociceptive pain. Sometimes more than one medication is needed to achieve pain relief [7,8,9]. Although persistent pain is reported more often by seniors living in nursing homes than by persons living independently [10,11], recent studies demonstrate that there is no association between chronic pain and cognitive or functional status. Perhaps pain is not a feature of aging, but it may contribute to functional deterioration [12].

Sometimes, there are mixed pain syndromes that include nociceptive and neuropathic pain, such as cancer-related pain. Chronic diseases related to neuropathic pain, such as diabetes mellitus, are very common in the general population. Moreover, aging is associated with a persistent inflammation state that carries high susceptibility not only to chronic morbidities but also to peripheral nerve sensitization. Considering that painful diabetic neuropathy affects one third of adults with diabetes mellitus [13], the prevalence of the neuropathic component of pain among older people should be higher than expected.

The pharmacological treatment of neuropathic pain in the elderly is often suboptimal [14]. Comorbidities may influence the correct management of chronic pain and its consequences among older people [3]. Some clinical conditions, such as chronic kidney disease or heart failure, require a careful evaluation of types, times and dosage of pharmacological therapies. Elderly, or persons generally affected by more than one illness, usually require chronic and multiple medications, which might interact with medication for persistent pain. This requires great attention from physicians; in fact, polypharmacy is associated with several adverse outcomes, including hospitalization, length of hospital stay and mortality. In a previous study, we described that polypharmacy (5–9 drugs) and excessive polypharmacy (≥10 drugs) are factors associated with polypharmacy status, including not only co-morbidity but also specific symptoms and age [15].

In literature, we classified the elderly into three groups: youngest-old, ages 65 to 74 years; middle-old, 75 to 84 years; and oldest-old, ≥85 years [16]. For the focus on very old people, who are often affected by malnutrition, sarcopenia and higher risk of falls, a different approach in the treatment of pain is required. In these cases, physicians should prefer lower dosages, alternative medications or nonpharmacological therapies.

Persons affected by neuropathic pain often report mood disorders and sleep disturbances as consequences of persistent pain. Lower satisfaction with life is common in patients with neuropathic pain, not only due to the symptoms of pain but also due to the impact of its consequences on the quality of life [17,18]. Neuropathic pain might affect the quality of life as much as other chronic illnesses, such as coronary artery disease or poorly controlled diabetes mellitus [19]. Depression is a common consequence, particularly if associated with higher pain intensity [17]. Concerning the elderly, untreated persistent pain is associated with poor sleep, social isolation, functional deterioration and increased risk of falls [20]. Anticonvulsants, such as pregabalin and gabapentin, as a first line therapy for neuropathic pain have are effective also for sleep disturbances.

## 2. Clinical Evaluation and Diagnosis

In order to choose the most appropriate treatment, it is important to know and identify the underlying mechanisms involved in pain perception (Table 1). Pain problems that arise from the stimulation of pain receptors give rise to nociceptive pain; generally, these receptors are stimulated as a result of trauma, inflammation and/or mechanical deformation. Examples may include ischemia, arthritis, infection, trauma and tissue distortion. Neuropathic pain results from pathophysiologic processes occurring in the central or peripheral nervous system. Some examples are diabetic neuralgia, posttraumatic neuralgia and postherpetic neuralgia. Central sensitization, a phenomenon resulting from synaptic plasticity, is important for the maintenance of chronic pain, both neuropathic and nociceptive. Increasing evidence suggests that this phenomenon is in part due to neuroinflammatory processes, involving both the peripheral and central nervous systems [21]. Moreover, there are other mechanisms of pain, including mixed nociceptive and neuropathic syndromes and pain syndromes of unknown mechanisms. Finally, it is important to consider the presence of psychological factors that may influence pain perception.

### 2.1. Clinical History and Common Symptoms

The most common conditions associated with neuropathic pain in the elderly are painful diabetic neuropathy, post-herpetic neuralgia, radiculopathies, post-traumatic neuralgia and central post-stroke pain. In older adults over 70 years of age, 3 out of 10 patients experience neuropathic pain [17]. However, while not a rare nosographic entity, it is often undiagnosed. The non-diagnosis, and therefore the failure to treat neuropathic pain, has important consequences for the health of the patient, especially the elderly. Because of the pain, the elderly patient can often experience depression, sleep disorders, falls, medication misuse, adverse drug reactions and slow rehabilitation. These complications, often persistent and coexisting, greatly complicate the initial diagnostic picture [22,23]. Moreover, older people may report less pain, often because they attribute pain to aging or do not report it for fear of losing independence or taking additional medication [24].

Neuropathic pain is generally a “persistent” (previously defined as “chronic”) pain, which therefore lasts for at least three months. In the diagnostic process it is particularly important to establish a pain history: characteristics, localization, triggering and relieving factors, onset, associated conditions or events that have occurred together with the pain and the treatments that have already been performed. Two symptoms are fundamental in neuropathic pain: allodynia (the perception of a harmless stimulus as painful) and hyperalgesia (the increase in painful perception of a painful stimulus) [25]. It is also important to identify a possible triggering factor, such as trauma (e.g., a fall, fracture or surgery), a recent acute disease (e.g., shingles), recent treatment (e.g., chemotherapy or radiotherapy) or any predisposing conditions such as a pre-existing disease (e.g., diabetes, neoplastic or rheumatic diseases). It is also important to take a family and social history, for example, to assess any psychological trauma (PTSD), the stability of ties with any partners and the use of tobacco, alcohol or other recreational substances. Elderly people can often have difficulty reporting or communicating pain, even in the absence of cognitive impairment [26]. Moreover, and especially in the elderly population, and in emergency period [27], it is important to assess any changes in behavior and to involve family members in the medical history collection in order to have a complete medical history. Finally, it is also important to keep in mind that nociceptive pain, if present, can mask neuropathic pain [28].

### 2.2. Physical Examination

During physical examination, an accurate and systematic neurological evaluation is important which assess any focal motor or sensitivity deficits; osteoarthritis; sarcopenia; gait alterations, reflex alterations that may indicate an alteration of the peripheral nervous system; alterations of the decubitus, such as the assumption of an analgesic posture or a defensive attitude towards a part of the body; and groans or paroxysmal cries that may indicate the presence of neuropathic pain [29,30,31,32]. In addition, it is important to look for skin alterations that may be a key sign (for example, the typical diabetic foot ulcer or dermatomeric distribution in shingles). Dysautonomic manifestations such as orthostatic hypotension, delayed gastric emptying or incontinence may imply that the pain is sustained by the autonomic sympathetic system or is a complex regional pain syndrome. Furthermore, especially in the elderly, an assessment of quality of life, functional status, psychological and social sphere to identify and treat anxiety, depression, social isolation and “disengagement” is a priority [33,34].

### 2.3. Pain Assessment

One way to categorize and quantify pain is using scales, especially in patients with cognitive impairment. Currently there are more than thirty scales to assess pain only in the elderly with cognitive impairment [28,35]. Although there are several scales that can be used to assess neuropathic pain, none have been universally approved for use in patients with advanced cognitive impairment. There are two main types of scales: one-dimensional and multidimensional, the latter generally give more stable values and explore more “domains of pain”. Some scales are specific for neuropathic pain, such as the Neuropathic Pain Scale (NPS) [36]. Other scales, although not specific for neuropathic pain, are particularly useful in the geriatric field when a patient suffers from advanced dementia, such as the Hurley Disconfort Scale (DS-DAT), which relies on the clinical presentation of the patient or self-report scales, such as the Verbal Rating Scale, the Horizontal Visual Analogue Scale and the Faces Pain Scale. All these scales have demonstrated reliability and stability over time. There are also questionnaires administrable to patients, such as DN4 [37] and painDETECT [38], which are designed to screen the subjective characteristics of neuropathic pain (such as burning, tingling, sensitivity to touch, pain caused by light pressure, electric shock-like pain, pain to cold or heat and numbness).

Recently, a system has been developed to classify the probability that a pain is neuropathic in poorly responsive patients: based on the presence of specific characteristics of neuropathic pain in the history, objective examination and confirmatory diagnostic tests, respectively, the pain is defined as “possible”, “probable” or “confirmed”. When a pain is classified as “probably” neuropathic, commencement of treatment is indicated [39]. Some confirmatory tests can be performed at the patient’s bedside, which include the evaluation of the presence of neuropathic pain characteristics, assessing the different components of sensitivity (touch, pressure, vibration, pain, temperature) and any alterations (eventually if there is a loss or an increase in somatosensory function). Neuropathic pain may begin with or without a harmful stimulating agent. In addition, more precise instrumental confirmatory tests such as quantitative sensory testing, blink reflex testing and the nerve conduction study can also be used. Furthermore, the diagnostic path of the elderly patient changes significantly depending on whether the patient is capable of self-assessment or not. Pickering et al. propose an algorithm that integrates the anamnestic research of possible causes of pain, comparing it with the physical objectivity and the use of specific questionnaires [40]. Finally, it is important for diagnostic and therapeutic purposes to identify the fragility of the elderly patient, both from a health and socio-economic point of view.

### 2.4. Main Etiological Scenarios

The main etiological scenarios can be classified depending on their origin as peripheral or central (Table 2).

- Peripheral.
  ○ Post-herpetic neuralgia (PHN);
  ○ Diabetic neuropathic pain (DNP);
  ○ Chemotherapy induced neuropathy (CIPN);
  ○ Post-operative neuropathic pain (PONP);
  ○ Complex regional pain syndrome (CRPS);
  ○ Compressive neuropathic pain (CNP);
  ○ Post-amputation neuropathic pain (PANP);
- Central.
  ○ Central post-stroke pain syndrome (CPSP);
  ○ Multiple sclerosis (MS);
  ○ Spinal cord injury (SCI);
  ○ Trigeminal neuralgia (TN).

#### 2.4.1. Peripheral Syndromes

##### Post-Herpetic Neuralgia (PHN)

The PHN is defined “pain continuing 90 days past the diagnosis of herpes zoster (HZ) or rash onset” [41]. The annual incidence of acute herpes zoster infection among healthy people under the age of 20 years is approximately 1 per 1000; the incidence is 5 to 10 times greater for those older than 80 years [42]. This also reflects the incidence of PHN in the elderly population [43,44]. Advanced age is therefore not only one of the main risk factors for the onset of PHN but is also associated with longer duration and severity of symptoms [45]. Usually the diagnosis is clinical, but in suspicious cases confirmatory laboratory tests are available (skin sampling, tissue biopsy, serology), although VZV DNA PCR has the highest sensitivity and specificity and has become the gold standard for diagnosis. In general, the symptoms and signs of posterpetic neuralgia (PHN) do not differ between the adult and elderly population and are mainly characterized by the triad of burning, sharp or stabbing and constant or intermittent pain and allodynia and anesthesia of the affected area. However, the early recognition of VZV reactivation is important in order to promptly start the specific treatment. This can be sometimes difficult, because in the elderly an atypical presentation compared to younger adults is possible; for example, it may appear as a patch inside the dermatormer or have a maculopapular appearance without vesicular evolution. After fading, the typical skin rash of the acute phase, initially a purplish–brownish discoloration, appears in the affected area, and then pale scarring results (scarring blades) appear. This scenario often evolves into an important painful condition, especially in the older people, and can also result in a loss of appetite, sleep and libido in the long term as this clinical scenario can last several months, risking a significant reduction in quality of life.

##### Diabetic Neuropathy (DNP)

Often, within ten years of the onset of diabetes, there is the onset of sensorimotor polyneuropathy [46]. When present, DNP causes several effects that significantly affect individual’s life as the pain significantly reduces the functional state of the patient, such as poor mobility and ability to walk [47]. The diagnosis is usually clinical, even if the presentation can be insidious, and is characterized by pain that is worsening at rest, typically during the night or in the early hours of sleep. This pain often also causes a reduction in the number of hours of sleep and the quality of sleep itself, which may lead to several complications in the elderly patient. The pain characteristics are shooting, stabbing or poking sensations with typical paroxysmal patterns; moreover, it can be associated with positive sensory alterations (the presence of burning, paresthesias or allodynia) or negative (loss of sensitivity) ones. The typical distribution is by socks and gloves. There are also other non-polar neuropathy sensorimotor diabetic forms (mononeuropathies, multiple mononeuropathies, plexopathies, autonomic neuropathies, etc.). Important differential diagnoses are claudication vasculopathy, Morton’s neuroma, radiculopathy, arthrosis, plantar fasciitis and tarsal tunnel syndrome; however, these have different clinical features and can be discriminated by imaging.

##### Chemotherapy Induced Peripheral Neuropathy (CIPN)

Chemotherapy-induced peripheral neuropathy (CIPN) is defined as somatic or autonomic signs or symptoms resulting from damage to the peripheral nervous system (PNS) or autonomic nervous system (ANS) caused by chemotherapeutic agents [48]. Common symptoms in CIPN are alteration in tattile, vibratatory, termic and nociceptive sensitivities, and the typical presentation is with painful paraesthesias in the extremities and signs consistent with an axonopathy. CIPN is rather important in older adults because it has been shown that those affected may display slower gait velocities, shorter step length and are at an increased risk of falls [49,50]. Older adults tend to develop chronic CIPN more frequently than younger adults [51]; this may lead to a functional decline and a higher risk of falls.

##### Post-Operative Neuropathic Pain (PONP)

Neuropathic pain resulting from operating procedures is the third leading cause of neuropathic pain in the elderly [17]. This type of pain has a complex nature, and it can be challenging to diagnose because of the variable manners in which older people can present it. Some studies show that pain could persist over a week for about one-third of post-operative patients [52]. This type of pain is more complex in older adults because of the interaction of potentially multiple factors, such as preexistent chronic pain, cancer and surgery itself, which in turn may alter the clinical presentation. Finally, recognition can be often complicated by post-operative delirium.

##### Complex Regional Pain Syndrome (CRPS)

The current accepted definition of CRPS is “an array of painful conditions that are characterized by a continuing (spontaneous and/or evoked) regional pain that is seemingly disproportionate in time or degree to the usual course of any known trauma or other lesion [53]. The pain is regional (not in a specific nerve territory or dermatome) and usually has a distal predominance of abnormal sensory, motor, sudomotor, vasomotor, and/or trophic findings” [54]. Pain is the main symptom, and it is described as burning, stinging or tearing. Although CRPS is a rare condition in the elderly, who have less inflammation after injury, it can be very debilitating, limiting individual functionality. The diagnosis is based upon clinical features, significant past medical history and physical examination. The main inciting events are traumas, surgery and fractures. Other important differential diagnosis are the presence of the Raynaud phenomenon, rheumatoid arthritis, diabetic neuropathy, deep vein thrombosis, compartment syndrome, peripheral vasculopathy and localized infection.

##### Compressive Neuropathic Pain (CNP)

Compressive radiculopathy is relatively common in the elderly because degenerative changes in the intervertebral disc occur typically with aging. Moreover, with aging, discs accumulate repeated mechanical stress over time, predisposing one to disk rupture and herniation. The diagnosis is both clinical and radiological [55]. The pain is the main symptom, but its characteristics varies depending on which nerve root is affected. The differential diagnosis of radiculopathy includes the peripheral nerve entrapment syndromes such as carpal tunnel syndrome, ulnar neuropathy at the elbow, etc. [56,57]. In these cases, a proper clinical evaluation associated with electrophysiological assessment and imaging (ultrasonography) provides useful data for diagnosis and treatment [58,59]. The onset of pain may be insidious or acute; the symptoms vary from a dull ache to a severe burning pain and differ in localization and distribution, but in general they follow the sensory nerve distribution. For example, in some cervical radiculopathy the patient may describe pain located on the medial border of the scapula and radiating to the proximal upper limb; similarly, some lumbar radiculopathy pain is referred in the buttock and radiating down in the lower limb. However, as already described, older patients may have some difficulties in describing pain localization, distribution and characteristics because of other co-existing medical condition, cognitive decline and language barriers, among others.

##### Post-Amputation Neuropathic Pain (PANP)

Although amputation is no longer a frequent procedure, when it is performed it most often causes chronic pain, which is reported in 95% of cases. Among all the causes of the amputation of the lower limbs, vascular diseases are particularly important, which have an increased incidence rate after the age of 65 years [60]. Amongst the causes of persistent pain after amputation, there are neuroma formation, phantom limb syndrome and flexion contraction. Neuroma can rise at any site of the peripheral nerve distribution. In phantom limb syndrome, pain is burning, aching or of a shock-type in the amputated limb and is the main symptom [61]. In addition to the significant pain of the phantom limb syndrome, related mechanisms are still little known [62]. There is also a long-term increase in the risk of developing depressive, anxious symptoms or significant post-traumatic psychological stress symptoms [63] that can adversely affect quality of life and facilitate cognitive decline. A flexion contracture can be a consequence of limb amputation. Such contracture is more common in older patients, especially if they also have cognitive impairment or have had a previous stroke. If the contracture becomes particularly important (over 25°) it can cause lumbar lordosis and low back pain.

#### 2.4.2. Central Syndromes

##### Central Post-Stroke Pain Syndrome (CPSP)

Central post-stroke pain syndrome is a condition characterized by the triad of allodinia, hyperpathia and sharp, stabbing or burning pain. This syndrome can commonly result from an ischemic lesion localized at any point along the spinothalamic or trigemino-thalamic pathways [64]. The site of the lesion affects the subjective localization of pain. The pain can be either caused or spontaneous and is often associated with dysesthesia [65], generally affecting large areas of the body, including the trunk and face; sometimes, it can affect narrower areas (e.g., a hand). Sometimes, it can manifest itself as unilateral facial and/or head pain. It is often an undiagnosed and therefore an untreated condition. Currently, the diagnosis is made by exclusion as no pathognomonic characteristics are evident [66]. Moreover, the diagnosis is often complicated by the fact that, especially in the elderly, pre-existing painful conditions are present and can be confusing in the clinical picture. Criteria have been proposed, divided into major and minor criteria, to facilitate and standardize the diagnosis of CPSP [66]. At this moment, we do not have studies that help in differential diagnosis. Advanced age does not seem to be a predictive factor of CPSP development after a cerebrovascular accident [67,68,69].

##### Multiple Sclerosis (MS)

Pain is often a common symptom in MS, although it is not always neuropathic in nature. Neuropathic pain can have a paroxysmal or persistent course, both of which are often associated with dysesthesia. Among the paroxysmal syndromes, there are trigeminal neuralgia, the anaconda sign and Lhermitte’s sign. Trigeminal neuralgia in MS (TN-MS) has similar presentation similar to non-MS population, but TN-MS is about 20 times more frequent than in non-MS population; it also tends to occur at an earlier age and often has a bilateral distribution. The anaconda sign, or the MS hug, is characterized by dysesthesias and an enveloping, tightening and oppressive sensation around the chest and abdomen, which occasionally can limit respiratory acts. Lhermitte’s sign is often elicited by flexion of the neck and is referred to as an electric shock radiating down the spine or into the limbs. Persistent pain can develop as a result of an increased frequency of paroxysmal pain attacks or directly with persistent pain syndromes, such as chronic migraine, pelvic pain syndromes, chronic tension-type headaches and atypical facial pain [70].

##### Spinal Cord Injury (SCI)

As a result of spinal injury, in about 60–70% of patients [71,72] a major painful syndrome, refractory to treatment and debilitating, may occur [73]. Nonetheless, the lack of a univocal definition of neuropathic pain subsequent SCI has already been highlighted as a major challenge. This syndrome is characterized by musculoskeletal, visceral and neuropathic pain, which significantly reduces the quality of life and sleep. Among them, neuropathic pain is the one that is most often reported as the most severe and excruciating [74]. Spinal cord stimulation devices should be considered in patient with chronic pain, included older ones [75,76]. Studies have shown that in elderly people with neuropathic pain from long-term spinal damage, it is associated with a higher incidence of depressive symptoms [77] and an increased use of health services, which tend to exacerbate the economic pressure on the health care system. The presentation of neuropathic pain in SCI is still poorly defined. Advanced age seems to be a risk factor for the development of neuropathic pain derived by spinal cord injury [78].

##### Trigeminal Neuralgia (TN)

Although primary trigeminal neuralgia is a rare condition, its incidence increases with age, so the TN is one of the more frequently seen neuralgias in the older adult population. It usually presents after the age of 50 but can occur at any age. In general, the manifestations do not differ in the elderly compared to the adult. The diagnosis is generally clinical and is mainly characterized by the presence of three main features of pain: unilateral localization (usually in one or more of the trigeminal branches), the sensation of a short and paroxysmal electric shock (<1–2 min of duration) and the ability to be triggered in response to innocuous stimuli; in particular, the latter feature seems to be the most specific of the syndrome [79,80]. Other possible diagnoses are to be ruled out, because they may mimic primary TN or may be secondary forms, such as trigeminal neuralgia related to herpetic or postherpetic manifestations, post-traumatic TN or other causes such as dental or craniofacial pain.

#### 2.4.3. Fibromyalgia

The definition and diagnosis of fibromyalgia has changed progressively over the past 40 years. At present, according to the IASP definition of neuropathic pain, it would not be included in this group of syndromes. However, recent studies have shown that there may be neuropathic small-fiber injuries [81], which would include, at the very least, fibromyalgia in neuropathic pain syndromes, influencing diagnosis and treatment.

## 3. Pharmacological Management

The management of neuropathic pain is based on a multidisciplinary team assessment [82], especially in the elderly, who are often affected by multiple diseases that require a more complex assessment than younger people. Pain relief and the improvement of the consequences derived by persistent pain should be the main goals for those who approach this issue. Guidelines for neuropathic pain management suggest a substantial drug approach with any distinctions based on different ages. In these circumstances, pharmacologic therapies represent the first step in pain treatment (Table 3).

Older people are often affected by more than one disease. Physicians should pay attention to drugs for chronic illnesses that have pharmacological interactions with pain medications. Metabolism in the elderly is compromised by physiological decrease in liver, kidney and heart function. Frailty and multimorbidity require a single-person strategy for pharmacological interventions. Starting with low doses and titrating very slowly are recommended. Some analgesic medications should be administered with caution in acute and long-term pain therapy. For example, NSAIDs (non-steroidal anti-inflammatory drugs) have several adverse reactions, including GI bleeding, renal impairment and platelet dysfunction; therefore, their use should be limited among older people.

Chronic pain is less manageable than acute pain. Patients affected by persistent pain should be aware that complete relief from neuropathic pain is difficult to achieve. For these reasons, pharmacological treatment often needs adjustments. It is necessary to review frequently the classes of medications, dosages, patterns and side effects to obtain an effective recovery. If a specific class of drugs is not efficacious, the use of an alternative one may be more favorable. Nonpharmacological strategies, such as cognitive behavior interventions, may be useful in combination with drugs or an alternative to them in long-term pain management. To avoid adverse effects of or addiction to the drugs, a nondrug intervention should be also considered as a bridge to other kinds of treatment.

Patients with persistent pain often experience sleep deprivation and mood disorders. Pain may lead to difficulties in initiating and maintaining sleep. As a result, sleep deprivation decreases the pain threshold and improves anxiety and depressed mood [82]. Nondrug assessment, including sleep restriction therapy, may help to improve the patient’s quality of life. Social isolation and loss of autonomy might complicate the pain assessement. Then, a mixed approach based on pharmacological interventions, cognitive behavioral therapies and rehabilitation is required. In the elderly, more so than in younger people, physiotherapists and occupational therapists assume an important role in the process of care.

### 3.1. Current Pain Medications

After excluding other kinds of pain or anatomical causes of persistent neuropathic pain that require a nonpharmacological approach, such as surgical treatment, physicians should focus on the right therapeutic strategy to obtain pain relief. Based on the actual guidelines, a substantial drug approach represents the first choice, especially among older people.

The main route of drug administration for neuropathic pain is the oral route. Considering that neuropathic pain is mostly a persistent pain, the oral route of drug administration is the most manageable. It gives an efficacious drug effect, which is prolonged over a specific period of time. Short-acting oral drugs are the most advantageous medications to take for a faster pain relief due to the rapid blood level onset for those forms of episodic pain. The intravenous route is preferable in the exacerbation of neuropathic pain when oral pills are not efficacious. The other routes of administration, such as transcutaneous, sublingual or subcutaneous are indicated when oral drugs are not efficacious or among the elderly with difficulty in swallowing [6,83].

#### 3.1.1. Anticonvulsants

Anticonvulsants, particularly gabapentinoids, represent the most effective class of drugs used for the treatment of neuropathic pain. Due to few drug interactions, gabapentinoids (pregabalin and gabapentin) are very manageable. Randomized trials on pregabalin and gabapentin have shown their effectiveness on peripheral neuropathic pain reduction. Gabapentinoids are also indicated in the treatment of postherpetic neuralgia, central neuropathic pain and the neuropathic component of cancer pain. Moreover, pregabalin improves the consequences derived by persistent pain, such as sleep disorders, depressed mood and social functioning [84]. As a “Level A” indication in the pharmacological treatment of neuropathic pain, pregabalin should be taken at a starting dose of 25 or 50 mg 2 times a day. At these doses, pregabalin should be effective on neuropathic pain among elderly patients, although the typical dosage effect starts at 150 mg/d [85]. Doses higher than 300 mg/d are typically associated with side effects. Although pregabalin’s quick titration is more tolerable than gabapentin, older people should assume a lower starting dose and increase the analgesic dosage with caution. Gabapentin should be titrated until two months, every seven days, to achieve a maximum tolerated dose. The starting dosage is 100 mg three times a day. At the beginning of titration, a single increased bedtime dose should be considered to avoid daytime sedation. The target gabapentin dose is between 1800 mg to 3600 mg/d [86], but it is necessary to evaluate renal function before increasing the daily dosage. Gabapentinoids may cause dizziness, diplopia, concentration disorders and peripheral edema, including ankle swelling. Sodium valproate is also effective on neuropathic pain, but its efficacy is probably less remarkable than pregabalin. It is associated with more side effects, such as poor glycemic control, haematological and gastrointestinal disorders. Carbamazepine is indicated as oral treatment only in persistent pain derived by trigeminal neuralgia. It is necessary to increase its dosage very slowly, considering the poor tolerability, with a starting dose of 200 mg a day. An alternative pain medication is oxcarbazepine, which has less pharmacological interactions and side effects than carbamazepine. Significant adverse reactions to carbamazepine are sleepiness, dizziness, ataxia, hyponatremia, SIADH, liver damage, aplastic anemia, leukopenia and thrombocytopenia. If carbamazepine and oxcarbazepine are both not tolerated, patients might take lamotrigine for a temporary period of time before conclusive surgical intervention.

#### 3.1.2. Serotonin Norepinephrine Reuptake Inhibitors (SNRIs)

Serotonin norepinephrine reuptake inhibitors (SNRIs), both duloxetine and venlafaxine, are approved for the treatment of painful diabetic neuropathy as an alternative therapy when gabapentinoids are not efficacious. Duloxetine should be taken at a starting oral dosage of 60 mg per day given in the morning. Among older people, a daily dosage of 30 mg/d could be also effective. It can cause nausea, vomiting, dizziness, somnolence, constipation and increased blood pressure. Duloxetine is also given in combination with gabapentinoids to decrease the threshold of neuropathic component of cancer pain. Venlafaxine is found to be effective not only in painful diabetic neuropathy but also in all the other form of painful polyneuropathies [82]. In the short-acting formulation, a dosage of 25 mg is given two or three times a day. Considering the long-acting venlafaxine, the starting dose is 37.5 mg or 75 mg once a day. The total daily dosage may be increased to 225 mg a day. Side effects, such as sweating, cardiac conduction abnormalities and high blood pressure are seen during the titration to the target dose. If a single-drug treatment is not effective for pain relief, adding venlafaxine to gabapentin may be more effective in the treatment of painful diabetic neuropathy [81]. This class of drugs shows its effectiveness not only on neuropathic pain but also on depressed mood, which is a common consequence derived by persistent pain.

#### 3.1.3. Tricyclic Antidepressants (TCA)

Tricyclic antidepressants (TCA), including amitriptyline, might be effective in painful diabetic neuropathy and postherpetic neuralgia. Nortriptyline is not less efficacious than gabapentin in postherpetic neuralgia. If a single drug approach does not give pain relief, considering that TCAs and gabapentinoids have different mechanism, a combination of these medication may be useful in management of neuropathic pain [86]. Considering the numerous adverse effects, such as cardiac conduction abnormalities, anticholinergic effects and postural hypotension, which can contribute to falls and fractures, TCAs do not represent the first pharmacological choice among older people.

#### 3.1.4. Opioid Analgesics

Opioid analgesics are considered a second line therapy in the treatment of acute or persistent nociceptive pain when common analgesic, such paracetamol e NSAIDs, are not efficacious. An association of opioid analgesics and the other classes of medications mentioned above represents the main pharmacological strategy to treat both the neuropathic and the nociceptive components of cancer pain. Opioid analgesics may be also useful in the management of exacerbation of persistent neuropathic pain. Considering that these drugs are associated with a prolonged half-life and longer duration of action among elderly, it is possible that a smaller than usual dose of opioids could be efficacious. Nevertheless, addiction to chronic use of opioids may represent an impediment to the treatment of persistent pain. Physicians should choose opioids only when alternative drugs are not efficacious. For example, dextromethorphan, tramadol, oxycodone and morphine sulfate are recommended, but with a “Level B” indication, in the treatment of painful diabetic neuropathy [84]. As a weaker opioid, tramadol is indicated in the treatment of painful diabetic neuropathy, trigeminal neuralgia and post-herpetic pain syndrome. The starting dose of tramadol is 50 mg once or twice a day. It is necessary to titrate tramadol very slowly, adding a daily oral dose of 50 mg every 7 days, until a total maximum dose of 300 mg a day is reached. Nausea, vomiting, seizures and orthostatic hypotension are common side effects. Adverse dose-dependent effects are somnolence, constipation and respiratory depression, which persist until the development of tolerance. During this time patients should pay attention to falls or other mobility accidents. Patients with renal or hepatic impairment should reduce the opioid dosage.

#### 3.1.5. Other Medications

An intravenous infusion of lidocaine, associated with rehydration, might be useful in the exacerbation of trigeminal neuralgia when the intensity of pain is very high and common oral drugs are not efficacious. An alternative to lidocaine is endovenous treatment with fosphenytoin [87]. Lidocaine might be also useful as a local anaesthetic when pain is well localized. Lidocaine patches should be applied directly on the painful site and be replaced every 12 h. If patches are not tolerated, lidocaine gel (6%) is an excellent alternative, especially on postherpetic neuralgia. Lidocaine patches might be helpful during titration of oral analgesic drugs. A combination of oral and topical treatment represents an alternative regimen to take pain relief. For other kinds of neuropathic pain, the effect of lidocaine is moderate.

High-concentration capsaicin (8%) patches show their effectiveness as a topical analgesic in the treatment of painful diabetic neuropathy and postherpetic neuralgia. The main side effect of these patches is the strong burning sensation on contact with warm fluid, which often requires an anaesthetic pre-medication.

Alternative medications for the treatment of neuropathic pain are cannabinoids, anti-arrhythmics, antioxidants (-lipoic acid), aldose reductase inhibitors, protein kinase C beta inhibitors and transketolase activators (thiamines and allithiamines), but their use is not common.

## 4. Surgical Therapies

Radiculopathy surgery, especially lumbar surgery, is currently considered a safe and effective intervention even in the elderly population [88]. Indeed, age is not an independent exclusion factor, but it is important to consider preoperative risk, which considers not only age and medical comorbidities but ensures a multidimensional assessment of the elderly. Efficacy, in terms of satisfaction and pain control after therapy, is similar both in the elderly and younger people. Given the increased prevalence of degenerative spinal disease associated with the aging of the general population, it is important to consider this type of treatment in the elderly as well [89].

Microvascular decompression is an effective procedure in the treatment of trigeminal neuralgia; this treatment has been shown to be as effective in the elderly as in the young. It is unclear whether it is this approach is riskier in the elderly than in the young; however, an increase in cases of death, stroke and thromboembolism has been noted in the former group [90].

Spinal Cord Stimulation (SCS) is an invasive neuromodulatory technique that should be considered in chronic pain that does not respond to conservative approaches and in particular when it is localized to one extremity. Its mechanism of action is still unclear. It has been hypothesized that it may regulate cytokines imbalance [75], but further studies are needed to ascertain that. At the state of the art, a multifactorial mechanism of action seems most likely. SCS has been shown to be effective in some types of chronic neuropathic pain, primarily in failed back surgery syndrome, but also in multiple sclerosis pain and complex regional pain syndrome. However, it may be less effective in postherpetic neuralgia and phantom limb syndrome [91]. Recent studies have shown how important it is to consider the complexity of pain and the possible overlap of multiple pain syndromes in the elderly patient when choosing a treatment [76].

More recently, dorsal root ganglia stimulation (DRGS) is being increasingly used as a first-line neuromodulation technique or in cases of SCS failure. Some data seem to show better outcomes than SCS, while maintaining comparable risks and complications of both techniques [92]. However, further studies are needed, especially in the elderly population to evaluate any differences in terms of efficacy and safety.

Nerve decompression surgery is a technique that aims to restore the function of compressed nerves. It has been hypothesized to be effective in some cases of diabetic peripheral neuropathy and superimposed focal nerve entrapment [92]; however, due to the presence of conflicting data, its use is not yet recommended for DNP [93].

Sympathetic nerve block (SNB) is a surgical technique that requires the presence of experienced staff and can achieve partially benefit in terms of reducing neuropathic pain. At present SNB is being used effectively to reduce pain in CRPS. It is not yet clear what the role may be in PHN [94]. In some cases, it has been hypothesized to be involved in DNP [95] and in PANP treatment [96].

Dorsal root entry zone (DREZ) has been described in the literature as a possible treatment for patients who have failed to respond to more conservative modes of therapy; however, at present, there are no studies indicating safety and efficacy in elderly patients.

## 5. Other Therapies

At the state of the art, pharmacologic therapies are the mainstay of neuropathic pain management; however, some nonpharmacologic therapies may be effective in adjuvating pharmacologic co-treatment [83]. The main non-pharmacological strategies include lifestyle modifications, physical therapies, surgery and microsurgery, cognitive-behavioral therapy and vaccines.

### 5.1. Lifestyle Modifications

Lifestyle modifications can be numerous and vary depending on the specific etiologic scenario; however, they are applicable wherever it is possible to correct an inappropriate behavior or an exposure to a modifiable risk factor, such that there is a significant impact in reducing the degree of pain, disease progression or quality of life.

### 5.2. Physical Therapies

Physical therapies that have demonstrated efficacy in the management of neuropathic pain include the application of superficial and deep level heat and cold, fluid therapy, whirlpool therapy, physical massage, TENS, transcranial magnetic stimulation and transcranial electrical stimulation.

Transcutaneous electrical nerve stimulation (TENS) has been shown to be one of the most effective physical therapies in the treatment of neuropathic pain; for instance, it is used to treat diabetic neuropathy that does not tolerate first-line therapies. Although there are not numerous studies, it has been shown in diabetic neuropathy to reduce pain [97]. In addition, TENS therapy has demonstrated effectiveness in treatment following spinal cord injury, acute, subacute, and chronic postoperative pain, and radiculopathy. Currently, the efficacy of TENS is believed to depend on intensity, frequency, duration and number of sessions. In elderly patients, when applied during exercise, TENS is well tolerated and can generate short-term hypoalgesia which may have beneficial short-term effects [98].

Transcranial direct current stimulation has been demonstrated that in some cases there is a reduction in pain intensity in the elderly [99,100]; however, its use may be limited by practical and regulatory issues.

Transcranial Magnetic Stimulation (TMS) has been shown to be a safe procedure and may be effective in some conditions [101]; studies have demonstrated pain reduction in chronic unilateral neuropathic pain from thalamic stroke, brainstem stroke, spinal cord lesion, brachial plexus lesion or trigeminal nerve lesion [102].

### 5.3. Rehabilitation

Rehabilitation is a widely employed element in the management of neuropathic pain [6]; the goal of this therapy is to adjuvate the pharmacological treatment, potentiating it, reducing the dose necessary to achieve the effective analgesic effect and improving the functionality and quality of life of the subject [103]. Although a fundamental element of rehabilitation is physical exercise, there are still no conclusive data of its effectiveness in neuropathic pain; however, the most studied area is diabetic and pre-diabetic neuropathy. Further research is needed to understand the role of exercise in sensory nerve disorders. The effectiveness of exercise depends on the type of underlying neuralgia [104,105].

### 5.4. Acupuncture

It has been demonstrated that acupuncture can be an effective therapeutic option in reducing the pain of diabetic neuropathy and chronic low back pain; however, it is still unclear how effective it is, even though safe and generally well tolerated, in other neuropathic pain syndromes such as post-stroke and post-herpetic neuralgia [96]. Acupuncture has been shown to be safe in many studies, although its efficacy compared to drug therapy has not yet been unequivocally demonstrated [106] and in some cases has proven ineffective. In elderly patients, it appears to be a good adjuvant therapy during the rehabilitation phase following acute disease, improving pain, quality of life and sleep and overall well-being [107].

### 5.5. Cognitive Behavioral Therapy

Cognitive behavioral therapy is used in the treatment of several conditions, both in young and older adults; the strongest evidence concerns anxiety disorders, somatoform disorders and bulimia. In addition, generalized anxiety disorder is not uncommon in the elderly [108]. Therefore, over the years more and more attention has been paid to the role and importance of psychological and social factors in chronic pain. This has contributed to the development of approaches such as cognitive behavioral therapy. Although the efficacy of this approach in the treatment of neuropathic pain is not universally validated, it is believed that given its low cost and safety, it is still a viable alternative in the management of neuropathic pain.

### 5.6. Varicella-Zoster Virus Vaccine

Age is the major risk factor for the development of herpes zoster and postherpetic neuralgia. The vaccine reduces the risk of herpes zoster incidence in the elderly population [109]. In addition, it generates a cell-mediated immunity response comparable to exposure to the Varicella-Zoster virus itself, which in turn is associated with a lesser severity of the course and incidence of postherpetic neuralgia [110].

## 6. Conclusions

A multidisciplinary team assessment represents the best strategy to manage neuropathic pain, especially among elderly, in those whom biological changes may alter not only the perception of pain but also the response to medications. A correct process of diagnosis requires an articulated anamnesis, which investigates comorbidities, consequences to chronic pain and previous pain medications. Questionnaires might help when older people are affected by cognitive impairments. Medications are more effective than non-pharmacological therapies, but often it is necessary to combine them, especially when neuropathic pain is not responsive to drugs. Rehabilitation and cognitive behavioral therapy represent an alternative regimen in the management of symptoms related to pain. Mostly, neuropathic pain is a persistent pain in which therapies are less efficacious than in nociceptive pain. Thus, particularly among older people who suffer from many diseases and often feel abandoned, physicians play a fundamental role in supporting patients in the whole process of care.

## Figures and Tables

**Table 1:** Pain pathomechanism.

Type of Pain	Nociceptive Pain	Neuropathic Pain	Central Sensitization
**Pathological mechanism**	Results from trauma, inflammation and/or mechanical deformation	Central or peripheral nerves damage	Absence of any nerve damage, trauma or inflammation
**Common syndromes**	Osteoarthritis Rheumatoid arthritis Tendonitis Neck pain Back pain Inflammatory disease	Diabetic neuropathic pain (DNP) Post-herpetic neuralgia (PHN) Chemotherapy induced neuropathy (CIPN) Post-operative neuropathic pain (PONP) Complex regional pain syndrome (CRPS) Compressive neuropathic pain (CNP) Post-amputation neuropathic pain (PANP) Central post-stroke pain syndrome (CPSP) Multiple sclerosis (MS) Spinal cord injury (SCI) Trigeminal Neuralgia (TN)	Chronic fatigue syndrome Fibromyalgia Restless leg syndrome

**Table 2:** Schematic representation of peripheral and central neuropathic pain.

Peripheral Neuropathc Pain
Post-herpetic neuralgia (PHN)
Diabetic neuropathic pain (DNP)
Chemotherapy induced neuropathy (CIPN)
Post-operative neuropathic pain (PONP)
Complex regional pain syndrome (CRPS)
Compressive neuropathic pain (CNP)
Post-amputation neuropathic pain (PANP)
**Central Neuropathic Pain**
Central post-stroke pain syndrome (CPSP)
Multiple sclerosis (MS)
Spinal cord injury (SCI)
Trigeminal neuralgia (TN)

**Table 3:** Pharmacological management of neuropathic pain.

Anticonvulsivant	Starting Dose	Elderly Point
Gabapentin	100 to 300 mg once to trice/day p.o.	Increased risk of serious, life-threatening and fatal respiratory depression and accidental injuries (e.g., falls).
Pregabalin	25 to 150 mg/day in 2 to 3 divided doses	Initiate therapy at the lowest dose. Increased risk of serious, life-threatening and fatal respiratory depression and accidental injuries (e.g., falls), visual impairment.
Carbamazepine	200 to 400 mg/day	Beers Criteria: potentially inappropriate medication, use with caution. Causes or exacerbates SIADH and hyponatremia. Increased risk of psychiatric effect; may activate latent psychosis, confusion, or agitation.
**SNRIs**		
Duloxetine	60 mg once daily	Beers Criteria: potentially inappropriate medication, use with caution. Causes or exacerbates SIADH and hyponatremia. Increased fall risk, with serious consequences. Antidepressants are associated with an decreased risk of suicidal ideation and suicidal tendencies in older adults ≥65 years of age.
Venlafaxine	37.5 mg or 75 mg once daily	Beers Criteria: potentially inappropriate medication, use with caution. Causes or exacerbates SIADH and hyponatremia. Increased risk of blood pressure elevation. May be associated with an increased risk of bone fractures. Antidepressants are associated with a decreased risk of suicidal ideation and suicidal tendencies in older adults ≥65 years of age.
**TCAs**		
Nortriptyline	10 to 25 mg/day	Beers Criteria: potentially inappropriate medication, use with caution. Causes or exacerbates SIADH, hyponatremia, sedation and orthostatic hypotension.
Amitriptyline	10 to 25 mg once daily at bedtime	Beers Criteria: potentially inappropriate medication, use with caution. Causes or exacerbates SIADH, hyponatremia, sedation and orthostatic hypotension. Antidepressants are associated with a decreased risk of suicidal ideation and suicidal tendencies in older adults ≥65 years of age.
